# Supplementary material for: A qualitative study of abortion professionals’ perspectives on doula support
Source: PLoS One. 2025 Dec 2;20(12):e0336290. doi: 10.1371/journal.pone.0336290 (PMC12671779; doi:10.1371/journal.pone.0336290)
Supplement: Attachment — Submitted filename: Review.docx [file pone.0336290.s001.docx]

Dear Authors,

Thank you for submitting your manuscript, "A Qualitative Study of Abortion Professionals’ Perspectives on Doula Support," for review. Your work addresses an important and underexplored topic in abortion care, particularly within the Canadian context. Below, I outline several key points for your consideration to strengthen the manuscript further:

Clarity in Sampling and Participant Demographics While the use of purposive and snowball sampling is noted, additional details about the selection criteria (e.g., specific roles, prior experience with doulas) would enhance transparency.

Including demographic data (e.g., age, years of experience, cultural background) for participants could help contextualize their perspectives and potential biases.

Methodology and Data Collection The shift from individual interviews to focus groups is understandable but warrants further discussion. For example:

Were there differences in themes emerging from focus groups versus individual interviews?

How were power dynamics or dominant voices managed in focus groups, given the sensitive topic?

Clarify whether data saturation was achieved and how this was determined.

Thematic Analysis Process Provide more detail about the coding process: How were initial codes developed? Was inter-coder reliability assessed? Were any software tools used (e.g., NVivo)?

Consider including a table or figure summarizing themes/subthemes with illustrative quotes for clarity.

Address whether participants were given the opportunity to review transcripts or findings (member checking).

Balancing Perspectives The manuscript highlights challenges in integrating doulas but could better acknowledge positive experiences or successful models (e.g., the Indigenous support program mentioned briefly). Are there lessons from these examples?

While the use of thematic analysis, as described by Braun and Clarke (Reference 30), is well-justified and widely accepted, the description of the collaborative coding process lacks sufficient detail to fully evaluate its rigor. Specifically:

Inter-Coder Reliability and Consistency: It is noted that two members conducted initial coding and developed a coding scheme, but the manuscript does not clarify whether these members:

Independently coded a subset of transcripts to establish inter-coder agreement (e.g., via Cohen’s kappa or percentage agreement). Collaboratively coded from the outset (and if so, how discrepancies were resolved).

Clarifying this process would strengthen the reproducibility of the findings. Refinement of the Coding Scheme: The manuscript mentions that the team adapted the coding scheme through discussion, but it would be helpful to specify: How many iterations of refinement occurred. Whether the final coding scheme was applied systematically by a single coder or multiple coders.

Limitations and Future Research The exclusion of patient voices is noted as a limitation, but its impact on the findings could be discussed further. For instance, how might patient perspectives alter the interpretation of "staff meeting patient needs"?

Suggest concrete future research directions, such as: Studies comparing clinics with/without doula integration. Patient experiences with doula support in Canada.

Structural and Editorial Refinements Some direct quotes lack sufficient interpretation. For example, the quote about "wrong information" (p. 15) could be tied more explicitly to concerns about doula training standardization.

Ensure consistency in terminology (e.g., "equity-deserving groups" is used but not defined).
