# Supplementary material for: A qualitative study of abortion professionals’ perspectives on doula support
Source: PLoS One. 2025 Dec 2;20(12):e0336290. doi: 10.1371/journal.pone.0336290 (PMC12671779; doi:10.1371/journal.pone.0336290)
Supplement: Attachment — Submitted filename: Response to reviewers.docx [file pone.0336290.s003.docx]

Dear Reviewer(s),

Thank you for your feedback and suggestions to improve the manuscript, “A Qualitative Study of Abortion Professionals’ Perspectives on Doula Support.” Please find below a point-by-point response.

**Reviewer 1:**

| **Comment** | **Response** | **Page/line number** |
| --- | --- | --- |
| Clarity in Sampling and Participant Demographics While the use of purposive and snowball sampling is noted, additional details about the selection criteria (e.g., specific roles, prior experience with doulas) would enhance transparency. | Added: All eligible and interested individuals were selected to participate and the research team sought to achieve diversity of professionals (physicians, nurses, social workers, clinic administrative staff) as well to obtain representation from across Canada. Prior experience with abortion doulas was not required. | 116-120, p. 5-6 |
| Including demographic data (e.g., age, years of experience, cultural background) for participants could help contextualize their perspectives and potential biases. | We did not collect demographic information due to the small size of this community and potential identifiability. This has been added in results, as well as included in limitations. | 159-161, p.7  390-392, p.18 |
| Methodology and Data Collection The shift from individual interviews to focus groups is understandable but warrants further discussion. For example: Were there differences in themes emerging from focus groups versus individual interviews?  How were power dynamics or dominant voices managed in focus groups, given the sensitive topic? | a) Added.  b) Added that they were all provided the option of individual interview or focus group. The sensitivity of the topic is familiar to participants given they are all healthcare professionals who work in abortion. During focus groups, the research team facilitated time for all participants to answer each question as well as the opportunity to provide private feedback later, at which point there were no concerns. | a) 139-140, p.6  b) 127-128, p.6 |
| Clarify whether data saturation was achieved and how this was determined. | Added: team determined conceptual depth, an alternative to data saturation described not as a “final limit, beyond which it is impossible to achieve new insights, but it is to reach a sufficient depth of understanding that can allow the researcher to theorise” through preliminary analysis of interview and focus group transcripts (citations added as well). | 124-128, p.6 |
| Thematic Analysis Process Provide more detail about the coding process: How were initial codes developed? Was inter-coder reliability assessed? Were any software tools used (e.g., NVivo)? | Thank you for this suggestion. More detail provided (per comment below as well). No specific software tools were used. | 132-139, p.6 |
| Consider including a table or figure summarizing themes/subthemes with illustrative quotes for clarity. | Added to table 1: Themes. | 163, p.7-8 |
| Address whether participants were given the opportunity to review transcripts or findings (member checking). | No, participants were not given the opportunity to review transcripts or findings as described on the consent form. Added. | 140-141, p.6 |
| Balancing Perspectives The manuscript highlights challenges in integrating doulas but could better acknowledge positive experiences or successful models (e.g., the Indigenous support program mentioned briefly). Are there lessons from these examples? | Thank you for this suggestion. We added a bit about the potential shift that this program could indicate, we have limited information about that program and of how the role of an Indigenous patient navigator differs from the role of an abortion doula. While the manuscript highlights challenges, we feel that the second theme: a place for abortion doulas, and the discussion also identify the potential benefits of their support and need for further exploration. | 363-366, p.17 |
| While the use of thematic analysis, as described by Braun and Clarke (Reference 30), is well-justified and widely accepted, the description of the collaborative coding process lacks sufficient detail to fully evaluate its rigor. Specifically: Inter-Coder Reliability and Consistency: It is noted that two members conducted initial coding and developed a coding scheme, but the manuscript does not clarify whether these members:  Independently coded a subset of transcripts to establish inter-coder agreement (e.g., via Cohen’s kappa or percentage agreement). Collaboratively coded from the outset (and if so, how discrepancies were resolved).  Clarifying this process would strengthen the reproducibility of the findings. Refinement of the Coding Scheme: The manuscript mentions that the team adapted the coding scheme through discussion, but it would be helpful to specify: How many iterations of refinement occurred. Whether the final coding scheme was applied systematically by a single coder or multiple coders. | Thank you for the suggested ways to make improvements.  Further detail added on independent coding, shared spreadsheet, and collaboration on final themes. | 132-141, p.6 |
| Limitations and Future Research The exclusion of patient voices is noted as a limitation, but its impact on the findings could be discussed further. For instance, how might patient perspectives alter the interpretation of "staff meeting patient needs"? | Good point, thank you – particularly given the difference between abortion doulas, and clinicians perspectives on the point. Added detail to limitations. | 395-398, p.18 |
| Suggest concrete future research directions, such as: Studies comparing clinics with/without doula integration. Patient experiences with doula support in Canada. | Added. | 398-400, p.18 |
| Structural and Editorial Refinements Some direct quotes lack sufficient interpretation. For example, the quote about "wrong information" (p. 15) could be tied more explicitly to concerns about doula training standardization. | Thank you for this feedback. Further interpretation added. | 177-197, p.9 |
| Ensure consistency in terminology (e.g., "equity-deserving groups" is used but not defined). | Added. | 74-75, p. 3-4 |

**Reviewer 2:**

| **Comment** | **Response** | **Page/line number** |
| --- | --- | --- |
| 1.Methods Transparency Sampling:  The shift from individual interviews to focus groups due to participant preference raises potential selection bias. Please: a) Specify how many participants were recruited via snowball vs. purposive sampling. b) Disclose any institutional affiliations (e.g., were all sites pro-choice organizations?) | a) added: 4/18 were via snowball sampling.  b) affiliation with Contraception and Abortion Research Team added.  Given study design participants work directly in abortion care whether as a clinical provider or social worker in clinic/hospital, or member of organization that facilitated access to abortion. Therefore, all would be considered pro-choice. | a) 160-161, p.7  b) 113-114, p. 5 |
| 2.Lack of Patient Perspective: The authors themselves acknowledge that patient voices are missing. Including patient experiences (or even those of doulas) would provide a more holistic view. | We agree, however this was outside the purview of the current study. Our previous research has explored the perspectives of abortion doulas as cited in the manuscript and we have added detail on suggestions for future research with patients. | 395-400, p.18 |
| 3.Over-Reliance on Clinic Settings: The emphasis on clinical concerns (space, workflow) may overshadow broader sociocultural dynamics or the ethical imperative for patient-centered support. | Thank you for this feedback. Added this to the discussion. | 381-383, p.18 |
| 4.Training and Certification Discussion Could Be Expanded: Given that "lack of standardization" is a key concern, it would be helpful to briefly explore efforts to professionalize doula training. | Thank you for this feedback. Added a sentence about this to the discussion with new citation. | 356-358, p.16-17 |
| The study makes an important contribution to reproductive health services research, particularly regarding equitable access for Indigenous and migrant populations highlighted in the results. | Thank you! | N/A |
